# Influence of the Manufacturing Process on Defects in the Galvanized Coating of High Carbon Steel Wires

**DOI:** 10.3390/ma10030264

**Published:** 2017-03-06

**Authors:** Marcello Gelfi, Luigi Solazzi, Sandro Poli

**Affiliations:** 1 Department of Mechanical and Industrial Engineering, Università degli Studi di Brescia, Via Branze 38, 25123 Brescia, Italy; luigi.solazzi@unibs.it; 2 IFP SpA, Via Predalva, 25050 Pian Camuno, Italy; poli@ifpwire.it

**Keywords:** hot-dip galvanizing, high carbon steel wire, cold drawing, defects, FEM, SEM, XRD

## Abstract

This study is a detailed failure analysis of galvanized high carbon steel wires, which developed coating cracks during the torsion test performed as a quality control at the end of the manufacturing process. Careful visual inspections showed that the cracks are already present in the coating before the torsion test. In order to explain the origin of these cracks, systematic metallographic investigations were performed by means of optical and scanning electron microscope on both the wires and the rods that have been cold drawn to produce the wire. The chemical composition of the galvanized coatings was evaluated by means of energy dispersive spectroscopy. Micro bidimensional X-ray diffraction experiments were also performed to measure the residual stresses in the galvanized coating. The results showed that the failure is related to two main factors: the relatively high content of silicon in the steel and the unsuitable cooling rate of the rods at the exit from the galvanizing bath. The mechanism proposed to explain the origin of the defects was supported by Finite Elements Methods simulations and verified with in-plant tests. The proper countermeasures were then applied and the problem successfully solved.

## 1. Introduction

**Figure 1:**
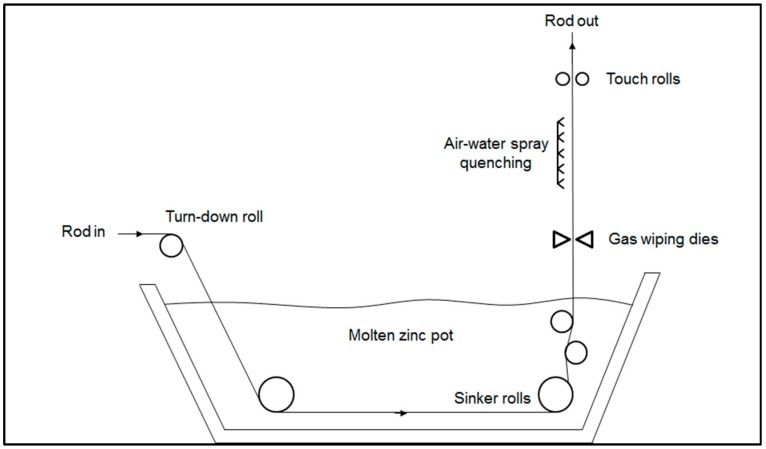
Schematic sketch of a continuous galvanizing process for wires and rods.

The manufacturing process of galvanized high carbon steel wires starts from the hot-rolled steel rods, which are austenitized in a furnace and then maintained in a lead bath at a temperature in the range of 520–560 °C. The soaking time in the bath is long enough to promote the isothermal transformation of austenite into a fine pearlitic microstructure, the so-called “patented microstructure”, which is ideal for the following cold drawing operations [1]. The lead particles remaining attached to the rod surface are removed by acid pickling, which is also a necessary surface pre-treatment for the following hot-dip galvanizing step. Before the immersion in the molten zinc bath, the steel rod is fluxed in an aqueous solution containing chloride salts to avoid any surface oxidation. The steel rod is then maintained in contact with the molten zinc at around 440–460 °C for a few minutes to form the galvanized coating on its surface [2]. At the exit from the molten zinc bath, the inert gas wiping dies accurately control the thickness of coating on the surface by removing the excess of molten zinc. Then the steel rod is spray quenched with a mixture of air and water to obtain a bright shiny finish [3] (Figure 1).

The zinc coating increases the corrosion resistance of the steel substrate [4] and it is a good solid lubricant to minimize the friction during the cold drawing process [5]. This process involves pulling the rod through a series of dies, with the diameter progressively reducing in size. At the exit of the last die, a small diameter wire (<5 mm) is produced with enhanced tensile strength properties due to the intense work hardening.

The as-fabricated galvanized steel wire needs to pass a number of quality tests before going on the market, concerning mechanical and corrosion properties. One of these is the torsion test that consists of counting the number of turns that the wire can sustain before failure.

**Figure 2:**
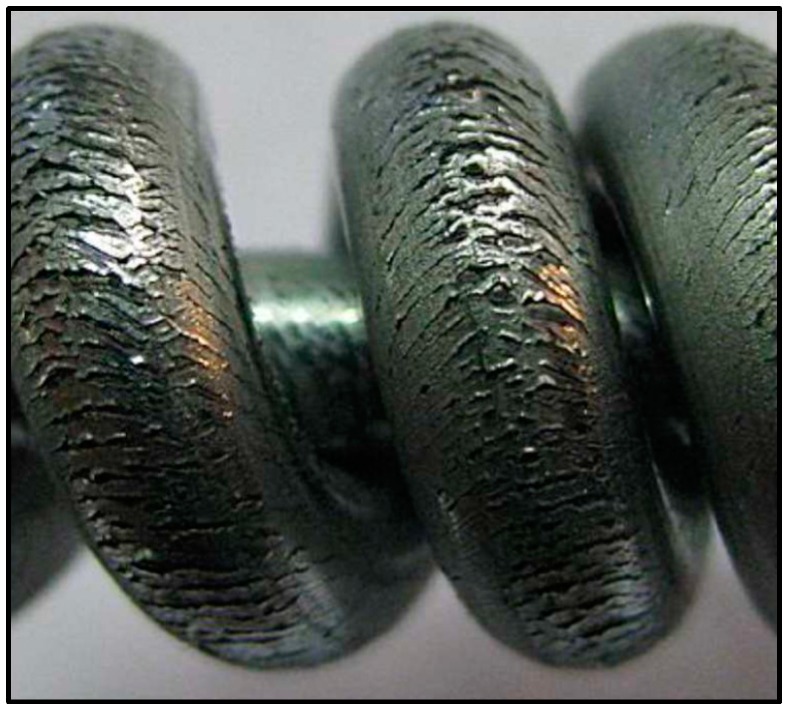
Circumferential cracks on steel wire after the torsion test.

In the present case, as reported by plant operators, the wire produced with specific steel batches at the end of the torsion test showed the formation of several circumferential cracks, randomly distributed along the circumference and length (Figure 2). A preliminary analysis showed that these cracks are limited to the Zn layers, suggesting that the coating could have some problems of adhesion or excessive brittleness.

In literature, the studies concerning the defects in galvanized coatings are mainly related to problems of delamination, also named “peeling off” or “shaving” of zinc coating, which happens during the forming operations. The main reasons that can produce this type of defect are the excessive plastic deformations [6], the abnormal growth of a brittle zeta phase in the coating [7,8], the presence of Mn oxide formed on the surface of the steel substrate during annealing before galvanizing [9,10].

To investigate the present problem, systematic metallographic investigations were performed on steel rods and wires by optical (OM) and scanning electron microscope (SEM). Micro bidimensional X-ray diffraction experiments (μXRD^2^) were also carried out to measure the residual stress in the coatings. A failure mechanism has been proposed also on the base of Finite Elements Methods (FEM) simulations that are normally used to model thermo-mechanical processes [11,12] and verified with in-plant tests.

Proper countermeasures have been adopted in the plant to solve the problem.

## 2. Experimental Procedure

### 2.1. Materials

The visual inspections of the wires belonging to the failed batch revealed the presence of fine circumferential cracks in the zinc coating also before the torsion test was performed. These cracks are difficult to detect by the naked eye, but they became evident when the torsion test is performed because they are enlarged by the applied shear stresses. To understand if the cracks are a consequence of the cold drawing process or if they come from the galvanized rod, both the wire and the rod have been analyzed.

The rods and wires considered in the work are made in C82D steel, according to the EN ISO 16120-2:2011 standard [13]. In particular, two different batches have been analyzed:

- Steel 1: C82D carbon steel rods and wires that failed during the torsion test;
- Steel 2: C82D carbon steel rods and wires that had no cracks.

The steel compositions of the two batches are shown in Table 1. All elements fit with the nominal ranges prescribed by the EN ISO 16120-2:2011 standard for the C82D steel. In both cases, the rod and the wire have a diameter of 7 mm and 4.5 mm respectively. 

The main parameters of the hot-dip galvanizing process are reported in Table 2.

### 2.2. Metallographic, Chemical and Residual Stress Analysis

Metallographic analyses were carried out on longitudinal and transversal cross sections cut from the rods and the wires in different positions. Samples were polished and etched with 2% Nital to reveal the microstructure of both coating and steel. The microstructures were examined using a LEO EVO-40XVP scanning electron microscope (Carl Zeiss Microscopy GmbH, Oberkochen, Germany). Semi-quantitative chemical analyses by the Link Analytical eXL microprobe (EDS) (Oxford Instruments NanoAnalysis, Buckingamshire, UK) were also carried out. 

The residual stress of the rods’ coating was evaluated by means of a D/max-RAPID Rigaku microdiffractometer (Rigaku Americas Corporation, The Woodlands, TX, USA) equipped with a cylindrical imaging plate (IP) detector by applying the DRAST method, which is based on a modified d-sin^2^ψ approach [14]. This system is suitable for performing residual stress measurements on metallic components with a relatively small size and/or a curved geometry [15], as in this case. Measurements were performed by using a beam collimator of 300 μm at a fixed incidence angle of 20°. The voltage and the current were set at 40 kV and 30 mA respectively, with an acquisition time of 1 h. The measurements were performed in the longitudinal direction by using the CuKα radiation. The residual stress was calculated for the outer eta layer by evaluating the distortion of the (101) Debye ring that is the strongest in this crystal lattice. The elastic constants of the eta bulk have been used for stress calculations: *E* = 75 GPa and *ν* = 0.30 [16].

### 2.3. FEM Simulation of the Defect

Numerical analysis by Finite Elements Method was applied to simulate the drawing of a galvanized steel wire. In particular, a small defect was considered to be present in the Zn coating, in order to study the deformation of both coating and steel bulk in the flawed area. The aim of this simulation was not to reproduce in detail the whole cold drawing process, but just to have qualitative information about the trend of the flawed area to assume the final defect morphology, i.e., the bulging of the steel substrate. For this reason, a single step of deformation has been considered. 

**Figure 3:**
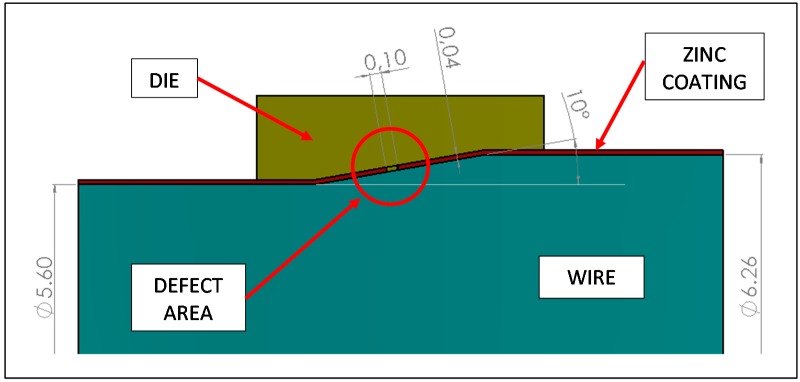
Model geometry for FEM analysis.

The model geometry defined for the simulation is shown in Figure 3. On the basis of metallographic analysis, the Zn coating thickness was fixed at 40 μm, while the defect was considered to be a coating delamination with the length of 100 μm. The approach angle of the die is 10° and the area reduction per drawing pass is 20%. The interface between the wire and the zinc coating was bounded, while the zinc coating and the die surfaces are in contact. The friction coefficient between the galvanized rod and the die wall was reasonably fixed at 0.1 [17].

The numerical analyses were performed by Autodesk Simulation^®^ software software (Software Release 2016, Autodesk Inc., San Rafael, CA, USA) and the FEM model used the axial-symmetry of the problem. The model is composed of about 700 thousand quadratic axial-symmetry elements. It is important to underline that the mesh is not uniform but the size of the elements is very small near the defect. In particular, in that zone, four rows of elements were created along the thickness of the zinc coating. A linear elastic model was considered to simulate the materials. The Young’s modulus, shear modulus and Poisson’s ratio of the steel substrate were taken to be 210 GPa, 81 GPa and 0.30 respectively, those of the coating layer were taken to be 140 GPa, 54 GPa and 0.30 [18]. The yield strength of steel was fixed at 1300 MPa considering an average strain hardening of 28% [19], while the yield strength of the coating was fixed at 130 MPa [18]. 

This is a complex problem due to the nonlinear behavior of materials and because different surface contact conditions have to be considered in the cracked area. For this aspect, many different numerical analyses were performed in order to evaluate both stress and strain in the wire and in the coating and to evaluate the deformation of the wire in the area not covered by the zinc layer. 

## 3. Results and Discussion

**Figure 4:**
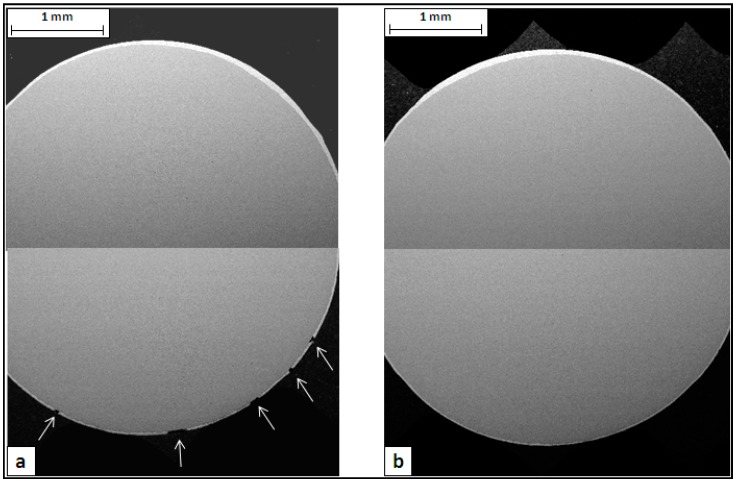
SEM images of wire cross sections after the torsion test: (**a**) Steel 1 and (**b**) Steel 2.

The metallographic analysis started considering the transversal sections of wires made with Steel 1 and 2 (Figure 4). The images, collected by SEM in backscattering mode at low magnification, show the zinc-rich coating as a thin white layer on the grey steel substrate. In both cases, it is evident that the thickness of the zinc layer is not uniform along the circumference. This is probably due to an excess of molten zinc that remained attached on the rod surface at the exit from the galvanizing bath. The analysis of rod cross sections showed the same uneven coating thickness, confirming this hypothesis. It is interesting to observe that in the failed wire, the defects are located only in one part of the cross section, where the coating is thinner (Figure 4a). 

**Figure 5:**
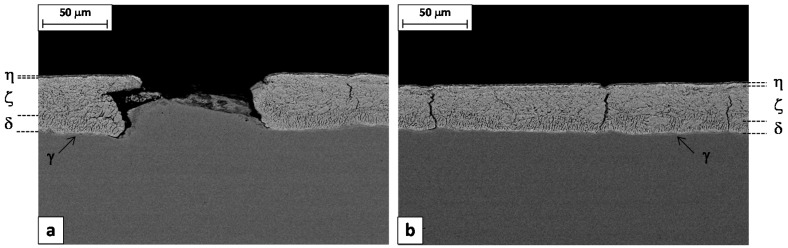
SEM images of Steel 1 wire sections: (**a**) defect cross section and (**b**) far from the defect and EDS chemical analysis of the bulged steel surface.

**Figure 6:**
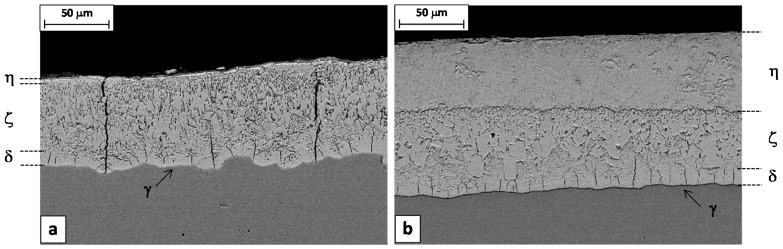
SEM images of Steel 1 rod longitudinal sections.

The metallographic analysis was then focused on the failed batch. Longitudinal sections were cut from both the Steel 1 wire and rod and etched by Nital2 to reveal the coating microstructure. SEM images were collected in back-scattering mode and reported in Figure 5 and Figure 6. Three layers can be clearly detected based on their iron content, measured by means of the EDS microprobe: eta (almost pure zinc), zeta (5%–6% wt. Fe) and delta (7%–11.5% wt. Fe) phase [2]. The gamma phase is also present as a very thin layer at the interface with the substrate. In any case, the lateral resolution of the EDS microprobe is not high enough to identify this phase separately from the others.

Figure 5a shows the longitudinal cross section of a defect responsible for the macroscopic failure of the wire during the torsion test. It consists of the local delamination of the Zn coating, which is missing for a length of about 100 μm. The steel substrate in the delaminated area is not flat but it shows a microscopic bulging. 

The residual zinc coating appears severely deformed by drawing, with the formation of several thin short cracks perpendicular to the substrate. Such cracks are confined to the delta layer, which is reasonable considering that this phase is more brittle than the others [20].

Long vertical cracks are also frequently present in the rest of the coating, at intervals of about 100–150 μm (Figure 5b). These cracks start from the interface with the substrate or from the gamma/delta interface and propagate along both the delta and the zeta layers, stopping when in contact with the eta phase that is soft and ductile.

It is interesting to observe that the length of the detached fragment is similar to the distance between two consecutive long cracks. Furthermore, in Figure 5a, one can observe that the left edge of the micro-bulged substrate fits almost perfectly into the cracked coating. These two observations suggest a possible mechanism for the formation of this kind of defect. It consists of two steps: (1) pulling the rod through the first drawing dies, a small fragment of the coating in between two long cracks is detached, (2) passing in the remaining dies, the steel substrate is coined in the “free space” remaining between the substrate and the die, giving the final appearance to the defect.

Figure 6a shows that the long vertical cracks are already present in the coating before the rod is drawn. In the region where the coating is thinner and the eta layer is almost absent, the cracks are able to pass the zinc coating completely. On the contrary, where the coating is thicker, the extra thickness consisting of the ductile eta layer avoids the propagation of the crack to the outer surface (Figure 6b).

This is in agreement with the previous observation that the defects are normally absent where the coating is thicker. The beneficial role of the ductile eta layer would be to mitigate the coating failure, sustaining cracked fragments of the brittle zeta, delta and gamma layers during the forming process.

**Figure 7:**
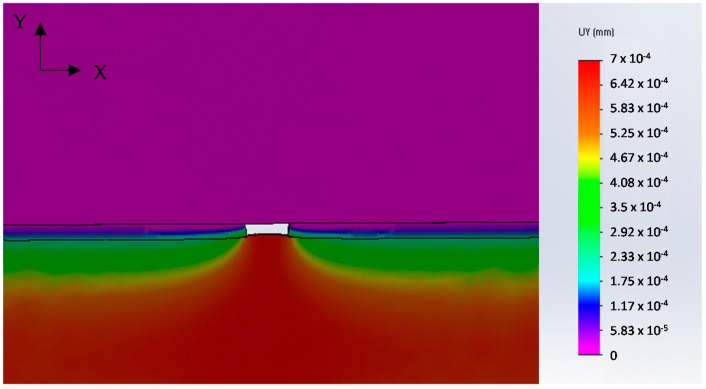
FEM simulations results: Y-displacements distribution.

Numerical analysis was applied to simulate the drawing of a galvanized steel wire containing the full detachment of the coating for a length of 40 μm. The results are presented in Figure 7 that shows the Y-displacements distribution in the area close to the defect. The tendency of steel substrate to protrude into the “free space” left by the coating is evident, in agreement with the morphology observed in Figure 5a.

This result supports the hypothesis that the origin of failure is the detachment of a coating fragment during the first drawing steps, favored by the presence of through-thickness cracks (Figure 6b).

**Figure 8:**
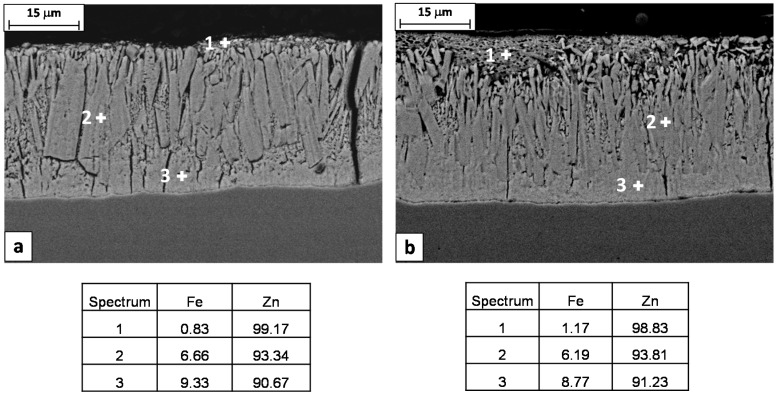
SEM images of longitudinal sections of (**a**) Steel 1 and (**b**) Steel 2 rods and EDS chemical analysis of the different coating layers (wt. %).

The presence of the vertical cracks in the hot dipped Zn coating is not surprising. The coefficient of thermal expansion of the coating layer is higher than that of the substrate steel, accordingly, the coating layer can suffer from multiple cracking during cooling, due to the thermally induced tensile stresses [18,21]. In any case, comparing the galvanizing coatings on the steel rods 1 and 2, it is evident that the through-thickness cracks are present only in the case of steel 1 (Figure 8). This can be explained considering that the two coatings have a different microstructure. In the case of steel 1, the columnar grains of the brittle zeta layer are coarser and the thickness of the soft eta layer is smaller compared to the steel 2 (Figure 8a). These conditions decrease the toughness of the coating, facilitating the propagation of cracks under the action of thermally induced tensile stresses.

The galvanized coating microstructure is strictly related to the chemical composition of steel. It is well known that in the process of hot-dip galvanizing, even small amounts of silicon in the substrate can increase the reactivity of the molten zinc. In steels with a content of Si of about 0.1 wt. %, the obtained coating exhibits the excessive growth of the intermetallic layers, resulting in poor mechanical and adhesion properties. This phenomenon is known as the Sandelin effect [22,23]. Also, P has synergetic activating effects on the reactivity of Si with molten zinc. Their conjoint effect is known as equivalent silicon (Si_eq_) and is defined as: Si_eq_ = %Si + 2.5%P. The recommended equivalent silicon for galvanizing steels is in the range of 0.14%–0.20% or less than 0.09% [6]. Beyond these limits, the coating has the tendency to be thicker, more porous and brittle.

The Steel 1 and 2 considered in this study have a Si_eq_ of 0.25% and 0.21% respectively. These values are both above what it is considered the optimal range for the galvanizing process. In particular, the higher value of Si_eq_ of Steel 1 can justify the different coating microstructure and its higher tendency for cracking.

The EDS chemical analysis of the coating layers also confirms that the Steel 1 has a higher reactivity with the molten zinc respect to Steel 2, showing higher contents of Fe in both zeta and delta phases (Figure 8).

Starting from these considerations, the high carbon steels processed in the plant were monitored to put in relation their chemical compositions with the frequency of cracks in galvanized wires. The results, reported in Table 3, show that a direct link exists between the failures and the silicon equivalent index. The wire made with a steel having the Si_eq_ index above 0.21% displays a high percentage of coating failures, while steels with a lower Si_eq_ index had no problems.

All these results confirmed that the formation of the multiple through-thickness cracks, responsible for the failure of the galvanized coating during the torsion test, depends on two main factors:

1. the intrinsic brittleness of the coating, when steel has the Si_eq_ index above 0.21%,
2. the high thermal tensile stresses developed during the cooling after galvanizing.

Avoiding this problem by controlling the Si_eq_ index always below the limit of 0.21% is probably not a viable solution, as silicon is an alloying element essential to improve the mechanical strength and the homogeneity of high carbon steels [24] or to promote the deoxidation of the melt during the steelmaking processes [25]. 

A better strategy could be trying to decrease the thermal stresses developed in the coating by reducing the cooling rate at the exit from the galvanizing bath. To test this approach, the rods made with the steel from the Batch 9 (see Table 3) were galvanized in two different ways:

- Steel 9A: by using the standard process;
- Steel 9B: by switching off the air–water spray at the exit from the Zn bath, in order to decrease the cooling rate.

**Figure 9:**
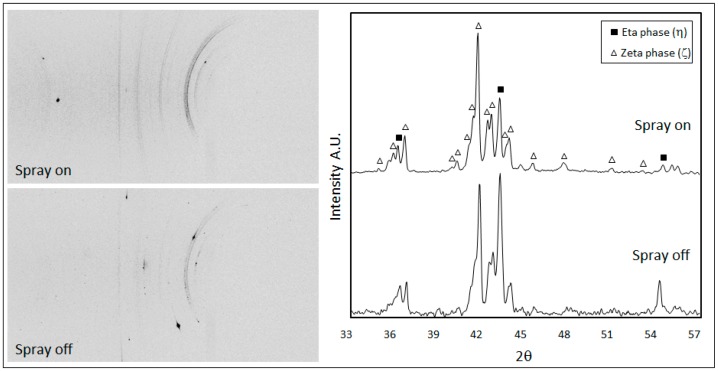
2D diffraction images and integrated patterns from steel 9A (spray on) and 9B (spray off) samples.

The longitudinal residual stresses were measured on these samples by means of μXRD^2^ technique. Figure 9 shows the 2D diffraction images collected from the samples 9A and 9B and the relative integrated diffractographs. According to the limited penetration depth of X-rays and the small incidence angle used in the experiments, only the diffraction peaks of the outer layers were detectable: the hexagonal eta phase (PDF#04-0831) and the monoclinic zeta phase FeZn_13_ (PDF#65-1238). In both samples, the Debye rings are not continuous but spotty, which means that the crystalline domains are rather large. This is true especially in the case of steel sample 9B, where the air–water spray was switched off giving more time to crystallites to solidify and grow. 

**Figure 10:**
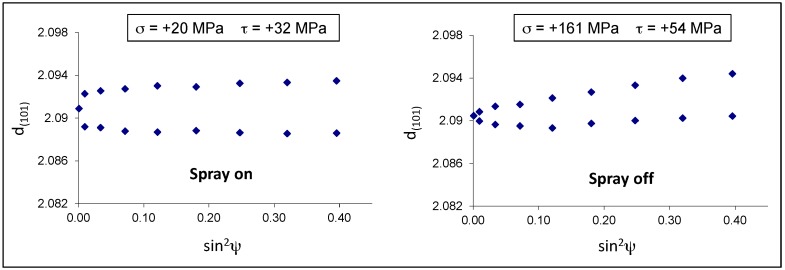
d-sin^2^ψ diagrams of steel 9A (spray on) and 9B (spray off) samples.

The residual stresses were calculated for the eta phase by measuring the deformation of the (101) Debye ring. Positive and negative ψ angles were considered. The d-sin^2^ψ graphs in both samples show an elliptical curvature, named ψ-splitting [26], due to shear stresses near the sample surface, probably related to interfacial stresses between the eta and zeta layers (Figure 10). It is interesting to observe that the air-cooled sample 9B developed normal tensile stresses (+161 MPa) much higher than those present in the spray-quenched one (+20 MPa). 

From a theoretical point of view, if the difference in thermal shrinkage between zinc and steel is fully accommodated by the elastic deformation of crystal lattice, then the misfit stress in the zinc coating will be more than 700 MPa at room temperature. However, works in literature showed that the actual residual stresses measured by XRD in the hot-dip zinc coatings are much lower, in the order of 100–200 MPa [9]. This is because the major part of the thermal misfit has been accommodated by plastic deformation of the zinc grains and/or by formation of microcracks along the zinc grain boundaries.

In the present case, the spray quenched sample shows residual stresses close to zero, probably because many microcracks developed in the eta layer and relaxed the tensile stresses almost completely. On the contrary, in the sample 9B, the slower cooling rate gave more time to accommodate the misfit between the coating and the steel by means of elastic and plastic deformation.

**Figure 11:**
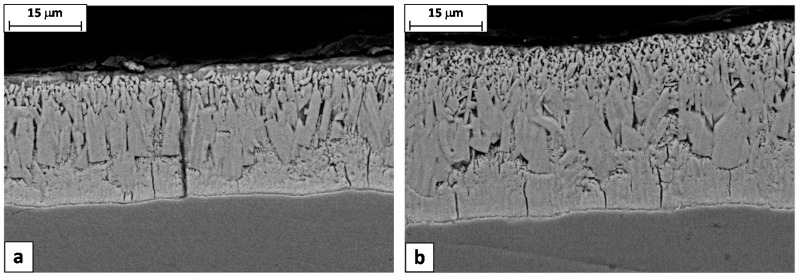
SEM images of longitudinal sections of (**a**) steel 9A and (**b**) steel 9B samples.

Longitudinal cross sections cut from the samples 9A and 9B confirmed this interpretation, showing that the through-thickness cracks are frequently encountered in the spray-quenched sample, while in the other one the cracks are always confined just in the delta layer (Figure 11). So, as expected, the soft cooling reduced the coating tendency to cracking. It is worth noting that the wire produced with the steel 9A had a high percentage of failures during the torsion test, while no failures were observed in the case of wire produced with the steel 9B. 

In conclusion, the proper countermeasure to prevent this type of failure was to reduce or completely switch off the air–water sprays when the Si_eq_ index of the rods’ steel exceeds the critical value of 0.21%.

## 4. Conclusions

This study showed that the cracks of galvanized high carbon steel wires developed during the torsion test are a consequence of preexisting through-thickness cracks in the rods’ coating. During the drawing process, from these weakened points, small fragments of the coating are detached, creating the final defects.

The presence of multiple through-thickness cracks in the rods’ coating depends mainly on two factors: the high thermal tensile stresses developed during the cooling down at the exit from the galvanizing bath and the intrinsic brittleness of the coating when the steel substrate has a Si_eq_ index above 0.21%. In this case, a coarser and more brittle microstructure is formed and, in particular, the thickness of the soft eta layer is almost zero, losing its ability to sustain the cracked fragments of intermetallics during the forming process.

In order to reduce the thermal stresses developed in the coating and the consequent cracks, in-plant tests were performed on steel rods with the Si_eq_ index above 0.21%, by switching off the air–water spray at the exit from the zinc bath. Measurements by micro X-ray diffraction showed that the reduction of cooling rate changed the residual stress state in the coating and the metallographic analysis confirmed the absence of through-thickness cracks. This solution completely solved the problem of coating failure in the wire.

In conclusion, it was proved that an effective countermeasure to prevent this failure consists of controlling the intensity of the air–water sprays accordingly to the Si_eq_ index of the steel rods.

## Figures and Tables

**Table 1:** Chemical composition (wt. %) of steel batches: Steel 1 (failed) and Steel 2 (good).

Steel	%C	%Si	%Mn	%S	%P	%Cu	%Ni	%Cr	Fe
EN ISO 16120-2	0.80–0.85	0.10–0.30	0.50–0.80	<0.030	<0.030	<0.25	<0.20	<0.15	Balance
Steel 1	0.81	0.215	0.705	0.004	0.015	0.053	0.022	0.03	Balance
Steel 2	0.827	0.188	0.663	0.008	0.01	0.022	0.019	0.032	Balance

**Table 2:** Hot-dip galvanizing process parameters.

Process Parameter	Value
Rod preheating temperature	160 °C
Molten zinc bath temperature	450 °C
Immersion time	≅10 s
Nominal bath composition	Zn 99.995%

**Table 3:** Relationship between steel composition and wire Zn coating failure.

Batch	Steel	%C	%Si	%P	Silicon Equivalent Index	Coating Failure Frequency
1	C82D	0.810	0.215	0.015	0.25	high
2	C82D	0.827	0.188	0.010	0.21	none
3	C76D	0.750	0.250	0.017	0.29	high
4	C72D	0.730	0.280	0.012	0.31	very high
5	C82D	0.835	0.183	0.010	0.21	none
6	C82D	0.833	0.190	0.006	0.20	none
7	C84D	0.840	0.190	0.009	0.21	none
8	C82D	0.810	0.270	0.016	0.31	very high
9	C82D	0.840	0.240	0.010	0.27	very high
